# Developing a reverse translational model of low-intensity rTMS in alcohol use disorder: The influence of theta burst stimulation protocols on binge alcohol drinking in mice

**DOI:** 10.1016/j.transm.2025.100098

**Published:** 2025-04-18

**Authors:** Akriti Dhungana, Daniel M. McCalley, Alesha M. Heath, Eric P. Kraybill, Fatemeh S. Mojabi, Jairelisse Morales Morales, Allison R. Morningstar, Allyson K. Davis, Claudia B. Padula, William J. Giardino, M. Windy McNerney

**Affiliations:** a Veterans Affairs Palo Alto Healthcare System, Sierra Pacific Mental Illness Research Education and Clinical Center (MIRECC), Palo Alto, CA, United States; b Stanford University School of Medicine, Department of Psychiatry and Behavioral Sciences, Stanford, CA, United States; c Stanford University, Department of Psychology, Stanford, CA, United States; d Stanford Wu Tsai Neurosciences Institute, United States.

**Keywords:** Transcranial Magnetic Stimulation, Alcohol Use Disorder, Brain Derived Neurotrophic Factor, Drinking in the Dark, Sex-differences

## Abstract

**Background:**

Repetitive Transcranial Magnetic Stimulation (rTMS) is a promising treatment tool for Alcohol Use Disorder (AUD). A challenge facing the field is that the optimal TMS parameters to reduce drinking are unknown. There are now rodent TMS coils which can be adapted to evaluate rTMS-induced changes in alcohol consumption in a rapid, cost-effective manner.

**Objective:**

Develop a preclinical model of rTMS in alcohol consuming rodents and collect pilot data to evaluate the influence rTMS parameters (here, theta burst pattern) on change in alcohol consumption and biochemistry.

**Methods:**

66 C57BL/6 J mice (32 F) received sham, intermittent Theta Burst Stimulation (iTBS), or continuous Theta Burst Stimulation (cTBS) (14 sessions, 2 sessions/day, low intensity 16mT stimulation). Alcohol consumption and preference were evaluated before and after rTMS using a two-bottle choice, Drinking in the Dark (DID) paradigm. Cortical brain tissue was assayed for BDNF gene expression via qPCR. During DID sessions, control mice (n = 31) were given access to water only.

**Results:**

Relative to sham, iTBS increased alcohol consumption (d=0.72) and preference (d=0.44), however these results were not statistically significant. Female mice receiving iTBS, experienced a significant, large increase in alcohol consumption (p = 0.02, d=1.5). Among water only mice, iTBS (d=−1.01) and cTBS (d=−1.03) significantly reduced BDNF expression.

**Conclusions:**

This preclinical model is a feasible method to evaluate rTMS-induced changes in alcohol consumption. This pilot analysis warrants future work evaluating the influence of rTMS parameters and sex on changes in drinking or drug-seeking behaviors.

## Introduction

Alcohol Use Disorder (AUD) levies an extraordinary burden on public health, with 5.1 % of disease and injuries worldwide directly attributable to alcohol use (Organization, 2018). Existing pharmaceutical and psychosocial treatment interventions are only modestly effective, with two thirds of individuals with an AUD relapsing to alcohol within 6-months of treatment (MATCH, 1997; Anton et al., 2006). Repetitive Transcranial Magnetic Stimulation (rTMS) is a promising non-invasive, therapuetic tool that can depolarize neuronal cell bodies in the induced electrical field and monosynaptic afferents. Clinically, patterned and repeated sessions of TMS (rTMS) can reduce relapse rates and alcohol consumption and craving (McCalley et al., 2022; Harel et al., 2021; Belgers et al., 2022; Padula et al., 2024; Mehta et al., 2024). However, a number of reports have demonstrated little to no change in alcohol drinking following rTMS (Hoppner et al., 2011; Herremans et al., 2015; Mishra et al., 2015, 2010). One reason for this variability in reductions to alcohol drinking and craving in the field likely stems from the use of a variety of rTMS protocols and parameter sets. For example, the chosen rTMS frequency (Huang et al., 2005; Pascual-Leone et al., 1994), number of pulses (Gamboa et al., 2010; McCalley et al., 2021; Nettekoven et al., 2014), number of rTMS sessions (Sackeim et al., 2020) or cortical target (Kreuzer et al., 2015; Hanlon et al., 2019) can all uniquely influence brain function and downstream changes in psychiatric symptoms. While some interesting patterns have emerged, such as delivering 10 or more sessions of rTMS tends to produce greater reductions in drinking (Mehta et al., 2024), rigorous exploration of this set of variables in the field remains a substantial challenge. Another, often over-looked factor influencing treatment response is biological sex. Among large-scale analyses of rTMS treatment response for depression, for example, individual sex has consistently been reported to influence treatment response, with females being generally more likely to experience response or remission from depression (Sackeim et al., 2020; Kedzior et al., 2014). While similar, sex-difference data from rTMS studies for AUD is limited, preliminary work suggests females with AUD experience greater rTMS-induced reductions in functional brain response to alcohol cues – a predictor of future relapse (McCalley et al., 2022; McCalley and Hanlon, 2021; Hanlon and McCalley, 2022). This work, however, is often challenged by generally lower census of females with AUD in clinical rTMS studies, reducing statistical power to detect sex-differences (Pinedo et al., 2020; Gunn et al., 2022). Taken together, there are two important scientific questions to address: 1) what is the optimal combination of rTMS parameters to reduce drinking in AUD patients and, 2) in prospectively balanced analyses, do sex-differences in treatment otucome for AUD (e.g. change in drinking) persist?

One strategy to fill these gaps is to develop preclinical, reverse translational models to evaluate the efficacy of rTMS parameters in reducing alcohol consumption within balanced samples of male and female animals. We and others have recently implemented rTMS coils previously developed to stimulate the cortex in awake, behaving mice (Madore et al., 2021; Makowiecki et al., 2014). Further, these coils can deliver high-frequency rTMS protocols that alter cognitive behaviors (McNerney et al., 2022) and expression of neuroplasticity-promoting proteins like Brain-Derived Neurotrophic Factor (BDNF) (McNerney et al., 2022, 2023). This feature of rTMS may be particularly relevant to AUDs, wherein BDNF is broadly reduced among patients (Silva-Pena et al., 2019). There are several additional advantages to evaluating the influence of rTMS parameters on drinking behavior in rodent models: 1) a wealth of translationally relevant models that can be used to explore changes in alcohol drinking, sensitivity, and conditioned reward (Thiele and Navarro, 2014; Cunningham et al., 2006; Crabbe et al., 2006), 2) preclinical studies can progress much faster and at a fraction of the cost of their clinical counterparts, and 3) preclinical models can allow for rigorous analysis of neurobiological mechanisms through which rTMS induces changes in gene and protein expression (Volz et al., 2013).

Here, our primary objective aimed to develop a reverse translational animal model of low-intensity rTMS among alcohol consuming mice. Using the preliminary data generated by the construction of this animal model, secondary goals of this manuscript were to evaluate the efficacy of 14 sessions of two common rTMS protocols, continuous and intermittent theta burst stimulation (TBS) or sham on drinking, and to explore sex differences in these metrics among male and female C57BL/6 J mice. With a balanced sample of male and female animals, we also aimed to compare sex differences in drinking and response to rTMS to expand the knowledge base built upon with clinical research. Here, we implement low-intenisty rTMS, as most rodent coils are too large to achieve focality. Low intensity coils, however, have higher focality and have been shown to induce changes in neural excitability (Tang et al., 2016). Research in the clinic is also beginning to explore the value of low-intensity stimulation (Castillo-Aguilar et al., 2021; Wu et al., 2024), warranting the need for preclinical research with low-intensity stimulation. In order to boost the ecological validity of this study, rTMS was delivered to awake-behaving mice and alcohol consumption was measured using a Drinking in the Dark (DID) paradigm, wherein mice were given the opportunity to freely consume ethanol and water in daily sessions before and following rTMS. Cortical BDNF gene expression following rTMS protocols was measured using quantitative PCR (qPCR). Our model presents a critical framework wherein the influence of rTMS parameters and sex on change in alcohol consumption can be rigorously and rapidly evaluated. Notably, this model can be readily extended to preclinical models of substance use disorders and rTMS-induced change in drug-seeking behaviors more broadly.

## Methods

### Mice

C57BL/6 J mice (n = 66, 3 months old) were housed in a temperature and humidity-controlled red-light room with 12-hour light/dark cycle and ad libitum access to food and water. All procedures were carried out under approval from the Institutional Animal Care and Use Committee (IACUC) at the Department of Veterans Affairs, Palo Alto. C57BL/6 J was chosen as this strain is well characterized in the context of several preclinical alcohol consumption assays (Thiele and Navarro, 2014).

### Surgical procedures

As previous reported, surgery was performed to affix a 1 cm, plastic rod to each animal’s scalp (Madore et al., 2021). This procedure was performed to create a rigid scaffold for the circular rTMS coil to affix to during stimulation. Briefly, mice were anesthetized with isoflurane and a 2–3 cm midline longitudinal incision was made through the scalp. The periosteum was scraped, and the premade coil support was attached over the bregma skull suture using cyanoacrylate and was further reinforced with dental cement. Post operative recovery occurred for one week following surgery.

### Volitional alcohol consumption: drinking in the dark

To model rodent drinking patterns before and after rTMS procedures, we implemented a two-bottle choice version of the DID paradigm for assaying binge-like volitional alcohol consumption. As previously described (Thiele and Navarro, 2014), mice were offered free access to bottles containing water (H2O) and alcohol (10–20 % EtOH) for 3-hours/day during the dark cycle (rodent active period). This model has high translational relevance to human models of binge drinking and can induce blood-alcohol concentrations similar to those observed in episodes of human binge drinking (Thiele and Navarro, 2014).

Prior to alcohol drinking, mice underwent a series of habituation steps three hours into their dark cycle to match optimal voluntary drinking conditions. On the first day, mice were habituated to their individual drinking cages and bottles for one hour. On the second day, the mice habituated to their drinking cages, bottles, and the rTMS coil for two hours. On days three and four, the mice habituated to their drinking cages, bottles, and rTMS coils for three hours. Mice were then habituated to alcohol by ramping from 10 % to 15–20 % ethanol during 3-hour sessions on three consecutive days. During this time, the amount of alcohol consumed was measured to determine the optimal concentration that would result in each mouse consuming approximately 3–4 g/kg, as recommended in previous publications to model moderate drinking with minimal changes in liver enzymes (Pruett et al., 2020).

**Fig. 1.**
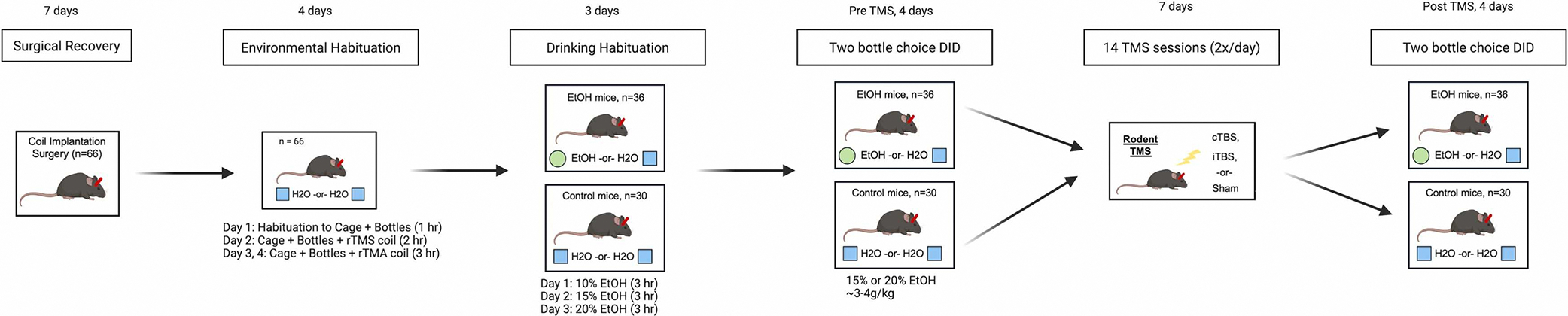
Experimental Design. Prior to experimental procedures, a plastic headpost was surgically affixed to the skull. 7 days following this surgery, mice were habituated to a novel cage context, ethanol, and bottles for two-bottle choice (3 days, 1 hour/session). Pre-TMS DID was assayed across the following 4 days (1 session/day, 3 hours/session). Mice were exposed to two bottles, containing ethanol and water (EtOH, n = 36) or two bottles containing water only (H2O, n = 30). Mice received 14 sessions of theta burst stimulation (2 sessions/day, 7 days). Followed by a 4-day, post-TMS DID period. Mice were euthanized and brain tissue was harvested for biochemical assays. Percentage of alcohol was calibrated for each animal to consume 3–4 g of ethanol/kg. The number of mice drinking 15 % or 20 % ethanol was not significantly different across TMS conditions (cTBS: 20 % EtOH, n = 6, 15 % EtOH n = 6; iTBS, 20 % EtOH, n = 6, 15 % EtOH n = 6; Sham 20 % EtOH, n = 8, 15 % EtOH n = 4).

Following habituation and prior to rTMS, mice (n = 36) were provided daily, 3-hour access to 15 % or 20 % EtOH and water (EtOH-H2O) for 4 days (one session/day). rTMS was then administered to mice twice daily for 7 days. Following rTMS, mice were again provided daily, 3-hour access given the same, individually-calibrated dose of alcohol as provided during pre-rTMS for 4 days (one session/day). Mice were euthanized via ketamine (80 mg/kg)/xylazine (10 mg/kg) overdose and underwent transcranial perfusion with saline the day after the final DID session. Brain tissue was extracted, and flash frozen for RNA extraction. As an experimental control for biochemical assays and to ensure rTMS did not induce broad effects on liquid consumption, 30 mice were given access to two bottles of water only while receiving the same handling and rTMS procedures as mice receiving ethanol. See Fig. 1.

### rTMS administration: intermittent and continuous TBS

rTMS was delivered using an in-house rodent rTMS coil (6 mm inner diameter, 8 mm outer diameter, 300 coil winding of copper wire) previously created by our collaborators and optimized for our experiments (Madore et al., 2021; Makowiecki et al., 2014; McNerney et al., 2022; Poh et al., 2018). Stimulation was controlled by LabView software through a LabJack T7 amplifier and connected to a custom power source. Alcohol-consuming mice were randomized to receive cTBS (EtOH-cTBS, n = 12, 6 F), iTBS (EtOH-iTBS, n = 12, 6 F), or sham (ETOH-Sham, n = 12, 6 F) stimulation. Water-consuming mice received cTBS (H2O-cTBS, n = 10, 5 F), iTBS (H2O-iTBS, n = 10, 5 F), or sham (H2O-Sham, n = 10, 4 F) following access to water only.

Theta burst stimulation parameters were chosen to match commonly used human protocols to the best of our ability (Huang et al., 2005). Two sessions of iTBS (600 pulses/session, bursts of 3 pulses at 50 Hz repeated at 5 Hz for a 2 s. train, with a 8 s. inter-train interval, pulse width 100us), cTBS (600 pulses/session, continuous bursts of 3 pulses at 50 Hz, repeating at 5 Hz, pulse width 100us), or sham (coil affixed to head mount, no active stimulation for 5 min) were delivered twice a day for 7 days. Stimulation began 3 hours into the dark cycle with a 1–1.5-hour gap between sessions. Active theta burst stimulation was delivered at an intensity of 16 mT (base of coil; estimated electrical field strength at cortex, 4 mV/mm (Madore et al., 2021)). rTMS was delivered in the same cage context as alcohol consumption, given reports that contextual cues related to alcohol and drug consumption during rTMS may amplify changes in alcohol and drug seeking behaviors (Dinur-Klein et al., 2014).

### BDNF expression

There is emerging interest in brain-derived neurotrophic factor (BDNF) as a biological substrate that mediates rTMS response (Bocchio-Chiavetto et al., 2008; Cheeran et al., 2008). We performed qPCR to evaluate the relative degree of BDNF gene expression following rTMS protocols in EtOH mice compared to H2O-only control mice. Cortical brain tissue samples were homogenized in Trizol and centrifuged at 12,000 xg for 5 minutes at 4 C. Chloroform was then added at one third of the supernatant volume and centrifuged at 12,000 xg for 5 minutes at 4 C. The RNA was precipitated using isopropanol and rinsed with EtOH. The RNA was resuspended in RNAse-free water with 1 % DNAse and quantified through nano dropping. Then, 1 μL of RNA was used to make cDNA (ThermoFisher 4368814). For qPCR, 2 μL of cDNA was used, in addition to 10 μL 2x Taqman Master Mix, 1 μL of primer (Taqman Mm04230607_s1; Mm999999_g1), and 7 μL of nuclease free water, to yield a total reaction volume of 20 μL, which was placed in a 96-well plate with samples in duplicates on a real-time qPCR cycler. The qPCR cycles were as follows: activation for 10 minutes at 95° C, amplification by 40 cycles of 15 seconds at 95 C, extension for 1 minute at 60 C. Standard curves and cycle threshold (Ct) values were generated, and the ddCT^2 was calculated using the housekeeping gene GADPH to determine relative quantities of gene expression for BDNF (Livak and Schmittgen, 2001).

### Statistical analysis

Data were analyzed using Prism 10.0 Software (GraphPad Inc., La Jolla, CA, USA). In order to analyze data from timepoints during which mice consumed stable, high levels of alcohol, we performed our primary statistical analyses for baseline alcohol consumption on the final two days of DID pre-rTMS. To match this timeframe, and to investigate the short-term effects of rTMS, we compared baseline alcohol consumption levels to those on the first two days of drinking following rTMS. To adjust for individual differences in baseline alcohol consumption, relative change scores in consumption and preference were calculated for each animal. Two-way ANOVAs were constructed with fixed effects for treatment type (cTBS, iTBS, sham) and sex (male, female), as well as a sex*treatment-type interaction term. For our ANOVA analysis of difference scores, we included Leven’s test for equality of variances and the Bruesch-Pagan test for heteroskedasticity, and neither of these indicated assumption violations (*p* = 0.20, *p* = 0.38). In a supplemental analysis, we created three-way repeated measures with fixed effects for time (pre-TMS, immediate 1–2 days post-TMS, and longer 3–4 days post-TMS), treatment type (cTBS, iTBS, sham) and sex (male, female) to evaluate changes in alcohol consumption and preference (see Supplemental Table 1). Leven’s test for equality of variances and Mauchley’s test for sphericity were not violated (p = 0.11, p = 0.34).

## Results

### Baseline ethanol consumption and preference

**Fig. 2.**
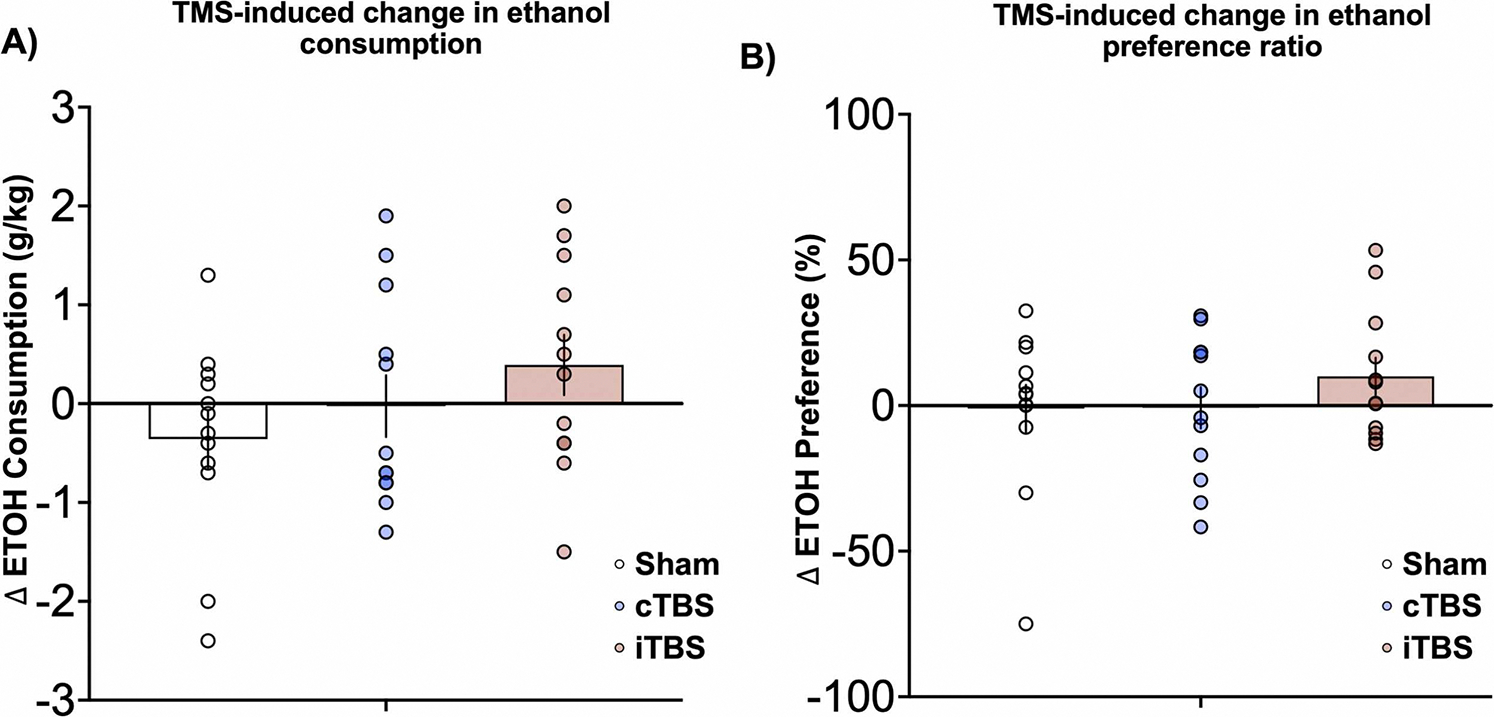
Relative change in alcohol consumption and preference for alcohol. A) Relative to sham stimulation (white), mice receiving cTBS (red) experienced a lesser decrease in ethanol consumption, while mice receiving iTBS (blue) experienced an increase in ethanol consumption (Change in ethanol consumption, average ± standard error of the mean; Sham, −0.35 ± 0.29 g/kg; cTBS: −0.025 ± 0.31 g/kg, p = 0.72, Cohen’s d=0.32; iTBS: 0.39 ± 0.30 g/kg, p = 0.2, Cohen’s d=0.72). There was no main effect of TMS treatment type (F_2,35_=1.53, p = 0.2). B) Mice receiving cTBS experienced relatively little change in ethanol preference, while mice receiving iTBS experienced a moderate increase in preference for ethanol (Sham, −0.99 ± 8.12; cTBS: −0.79 ± 7.10, p = 0.9, Cohen’s d=0.01; iTBS: 10.04 ± 6.4, p = 0.5, Cohen’s d=0.44). Error bars reflect standard error of the mean. Individual data are plotted in each circle.

Across four days of 3 hr two-bottle choice (baseline EtOH DID sessions prior to rTMS), mice averaged intakes of 2.23 ± 1.58 g/kg and 72.4 ± 20.3 % preference for alcohol, relative to water (Fig. 2A–B). Relative to males, females consumed significantly more ethanol (t_34_=3.6, p = 0.001, Cohen’s d=1.2) and significantly more water (t_33_=2.2, p = 0.03, Cohen’s d=0.7; Supplemental Fig. 1). However, there was no sex-difference in preference for alcohol (p = 0.87, Cohen’s d=0.05). There was no difference in the number of mice drinking 15 % or 20 % EtOH solutions across groups (χ^2^=0.9, p = 0.64).

### rTMS-related changes in alcohol intake and preference

There were no significant main effects of interactions within our 2-way ANOVAs evaluating change in alcohol consumption or preference (See Supplemental Table 1). To further evaluate longer-term changes in alcohol consumption and preference, we computed a 3-way (time, treatment, sex), ANOVA on ethanol intake and preference, including the final two days prior to rTMS, the first two days after rTMS, and days 3 and 4 after rTMS. With repeated measures ANOVA, we detected a main effect of sex (F_1,30_=9.05, p = 0.005) and a trend-level time*-sex*treatment interaction (F_4,60_=2.39, p = 0.06). There was not a significant main effect of time or treatment, nor a time*treatment, treatment*sex, nor time*sex interactions. See Supplemental Table 2 and Supplemental Fig. 2 for expanded information.

**Fig. 3.**
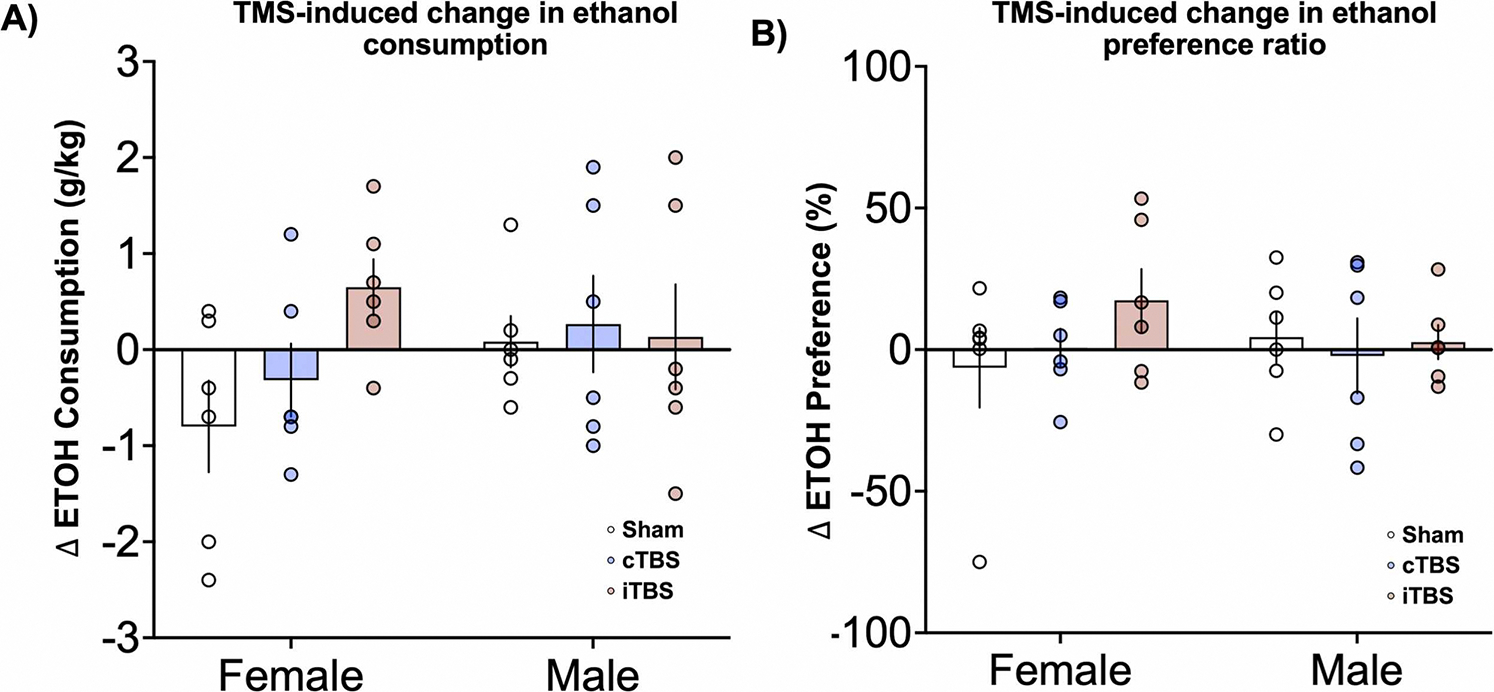
Sex differences in relative change in alcohol consumption. A) Relative to baseline pre-TMS levels, female mice (left side) reduced ethanol consumption following cTBS (blue) and increased ethanol consumption following iTBS (red) (Sham: −0.80 ± 1.16 g/kg; cTBS: −0.32 ± 0.93 g/kg, p = 0.4, Cohen’s d=0.45; iTBS: 0.67 ± 0.71 g/kg, p = 0.02, Cohen’s d=1.5). Male mice (right side), experienced increases in ethanol consumption following both cTBS and iTBS (Sham: 0.07 ± 0.65 g/kg; cTBS: 0.26 ± 1.23 g/kg, p = 0.7, Cohen’s d=0.19; iTBS, 0.13 ± 1.34 g/kg, p = 0.9 Cohen’s d=0.05). B) Relative to same-sex sham TMS mice, female mice experienced a no change in preference for ethanol following cTBS and an increase in ethanol preference following iTBS (Female mice, sham, −0.64 ± 34.4 %; cTBS, 0.6 ± 16.6 %, p = 0.60 Cohen’s d=0.04; iTBS: 17.4 ± 27.0 %, p = 0.11, Cohen’s d=0.44). Male mice experienced no change in ethanol preference following cTBS and iTBS (Male mice, sham: 4.4 ± 22.0 %; cTBS: −2.2 ± 32.5 %, p = 0.66, Cohen’s d=0.24; iTBS: 2.6 ± 14.84 %, p = 0.91, Cohen’s d=0.1). Error bars reflect standard error of the mean. Individual data are plotted in each circle.

Although there were few statistically significant changes in alcohol consumption within this pilot study, we identified several interesting qualitative rTMS-induced changes in alcohol drinking. While not statistically significant, relative to sham control mice and baseline pre-rTMS levels, iTBS increased overall EtOH intake (Cohen’s d=0.72, p = 0.20) and preference (Cohen’s d=0.44, p = 0.50) (Fig. 2A–B). Given our interest in sex differences and the effect in baseline alcohol and water consumption, we conducted exploratory analyses to evaluate sex differences following rTMS. Relative to same-sex controls and baseline pre-rTMS levels, iTBS significantly increased EtOH intake in female mice (Cohen’s d=1.5, p = 0.02) (Fig. 3A). cTBS trended toward increasing alcohol preference in female mice (Cohen’s d=0.44, p = 0.11, Fig. 3B). Among male mice, both cTBS and iTBS did not increase alcohol intake, relative to same-sex controls and baseline consumption levels (cTBS: Cohen’s d=0.19, p = 0.7; iTBS: Cohen’s d=0.05, p = 0.9; Fig. 3D). There were no changes in water consumption among water control (Supplemental Fig.3). Average alcohol intake and preference data are available in Supplemental Fig. 4.

### rTMS-related and alcohol-related changes in BDNF expression

**Fig. 4.**
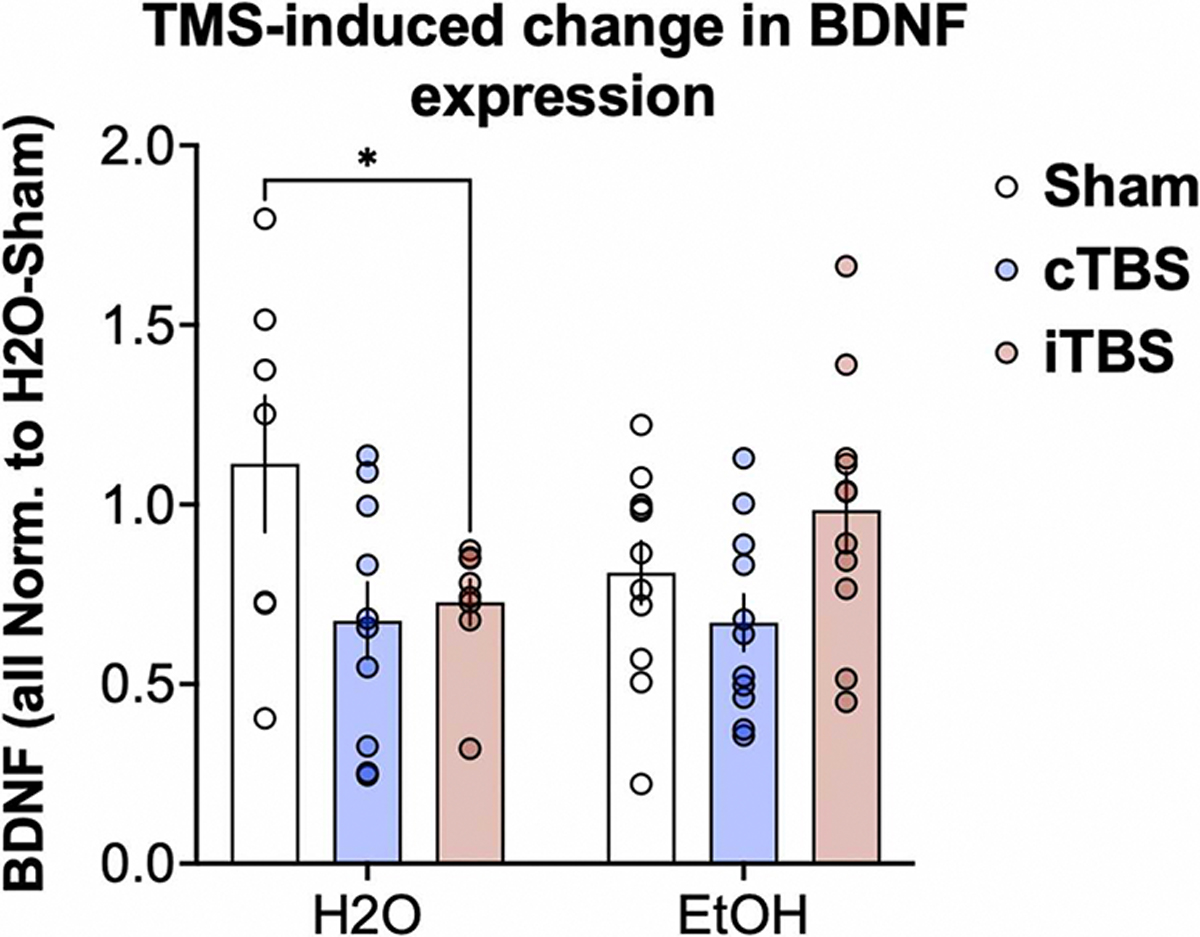
rTMS-induced change in BDNF expression. Among mice with access to water only (H2O), there was an overall decrease in BDNF expression following both cTBS and iTBS, relative to sham (Sham H2O: 1.11 ± 0.50; cTBS H2O: 0.67 ± 0.33, t_52_=2.72, p = 0.012, Cohen’s d=−1.03; iTBS H2O: 0.73 ± 0.18, t_52_=2.29, p = 0.03, Cohen’s d=−1.01). Mice who consumed ethanol and received sham rTMS experienced moderate reductions to BDNF expression, relative to sham rTMS, H2O mice (Sham, water: 1.11 ± 0.50; Sham, alcohol: 0.81 ± 0.29, t_52_=1.63, p = 0.12, Cohen’s d=−0.73). Relative to sham rTMS, EtOH mice, cTBS reduced while iTBS increased BDNF expression (Sham, EtOH: 0.81 ± 0.29; cTBS, EtOH: 0.67 ± 0.26, t_52_=1.0, p = 0.38, Cohen’s d=−0.51; iTBS, EtOH: 0.98 ± 0.35, t_52_=1.26, p = 0.38, Cohen’s d=0.52. Error bars reflect standard error of the mean. Individual data are plotted in each circle.

There was a significant effect of rTMS treatment type (F_2,52_=3.84, p = 0.03), as well as a rTMS treatment x liquid type interaction (F_2, 52_= 3.28, p = 0.04) on cortical BDNF mRNA gene expression levels. Among water-only control mice, iTBS and cTBS significantly reduced BDNF mRNA expression levels (iTBS: Cohen’s d=−1.01, p = 0.03; cTBS: Cohen’s d=−1.03, p = 0.01), relative to sham stimulation (Fig. 4A). There were no statistically significant effects of rTMS treatment among alcohol drinking mice. Qualitatively, BDNF expression levels were slightly reduced by cTBS and slightly increased by iTBS, relative to sham controls (cTBS: Cohen’s d=−0.51, p = 0.38; iTBS: Cohen’s d=0.52, p = 0.38; Fig. 4B).

**Fig. 5.**
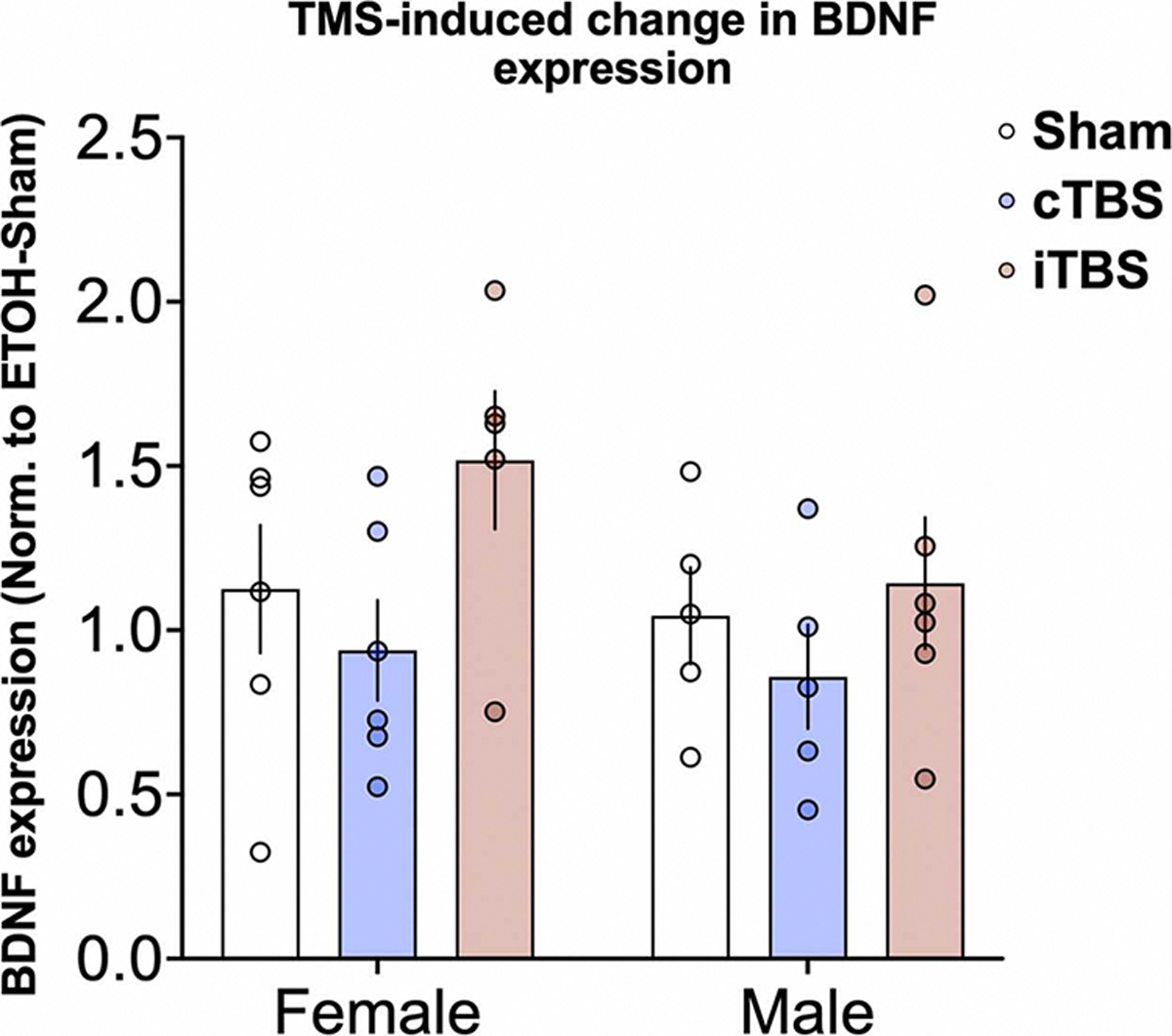
Sex differences in change in BDNF expression among alcohol drinking mice. Relative to same-sex mice receiving sham rTMS, female mice experienced a decrease in BDNF expression following cTBS and an increase following iTBS (Sham, female: 1.12 ± 0.48; cTBS, female: 0.94 ± 0.37, t_27_=0.69, p = 0.67, Cohen’s d=−0.42; iTBS, female; 1.52 ± 0.47, t_27_=1.53, p = 0.23, Cohen’s d=0.84). Male mice experienced a similar pattern, wherein cTBS reduced and iTBS increased BDNF expression (Sham, male: 1.04 ± 0.33; cTBS, male, 0.86 ± 0.35, t_27_=0.38, p = 0.71, Cohen’s d=−0.53, iTBS, male, 1.14 ± 0.49, t_27_=0.39, p = 0.90, Cohen’s d=0.23). Error bars reflect standard error of the mean. Individual data are plotted in each circle.

In a second level of inquiry, we analyzed sex differences in BDNF expression. While results did not reach statistically significant thresholds, we observed several intriguing patterns. Among alcohol drinking mice, iTBS qualitatively increased BDNF expression among females (Cohen’s d=0.84, p = 0.23), while there were no effects of cTBS on BDNF expression in males (p = 0.71) and females (p = 0.67) (Fig. 5). Qualitatively, this pattern was maintained when BDNF expression data were normalized to same-sex, H2O-consuming animals which received sham rTMS (Supplemental Fig. 5). We did not detect a significant relationship between BDNF expression and change in alcohol consumption or preference (all p’s > .05).

## Discussion

There is tremendous enthusiasm surrounding the development of rTMS as a therapeutic tool for Alcohol Use Disorder (Ekhtiari et al., 2019; Philip et al., 2020). While this field rapidly develops, it is critical to consider that rTMS itself is not a monolith – its efficacy is directly dependent on an array of parameters (e.g. frequency, number of pulses, number of sessions, stimulation strength) which with each likely to uniquely influence treatment outcome (Huang et al., 2005; Pascual-Leone et al., 1994; Gamboa et al., 2010; McCalley et al., 2021; Nettekoven et al., 2014; Sackeim et al., 2020; Kreuzer et al., 2015; Hanlon et al., 2019). There are also several reports demonstrating sex-differences in response to rTMS for AUD (McCalley et al., 2022; McCalley and Hanlon, 2021; Hanlon and McCalley, 2022), however clinical trials for AUD are rarely equipped to adequately assess sex-differences. Translational, preclinical models of binge drinking and AUD have the potential to develop into high value, rapid, and cost-effective resources that may be used to evaluate the influence of rTMS parameters and sex-differences on drinking. Here, our primary objective was to develop a preclinical model of rTMS in a sex-balanced sample of C57BL/6 J, alcohol-consuming mice (n = 66, 30 female). To demonstrate the potential utility of this technique, we evaluated the efficacy of two common rTMS protocols, continuous and intermittent theta burst simulation, to manipulate alcohol drinking, preference, and brain-based BDNF levels, relative to sham stimulation.

Our primary goal, to develop this preclinical, rTMS model of alcohol consumption, was motivated by a persistent challenge in the human literature – high degrees of variability in response to treatment. Several factors likely contribute to the observed variability in treatment outcome, including factors external to rTMS (e.g. genotype, brain state), as well as rTMS stimulation parameter choices (e.g. number of sessions, stimulation intensity, pattern of stimulation). The preclinical model developed here may be particularly advantageous in that it can be readily adapted to hold external biological and environmental conditions constant while directly evaluating various rTMS parameters. To advance the field’s knowledge regarding the influence of rTMS treatment parameters in a practical and translatable manner, our study procedures aimed to carefully replicate alcohol binge drinking patterns and rTMS delivery protocols in clinical settings. For example, we delivered rTMS to awake, behaving mice in the absence of anesthesia. During all alcohol access sessions, mice were free to choose between drinking water or alcohol, rather than artificially inducing high blood alcohol levels by forced experimenter administration. Notably, our chosen volitional drinking protocol in mice (two-bottle choice DID) has been shown to produce blood alcohol concentrations equivalent to the NIAAA definition of binge alcohol drinking in humans (Thiele and Navarro, 2014). Lastly, rTMS delivery to mice occurred in their same cage environment in which they received access to alcohol. This experimental design is analogous to studies from the human literature wherein exposure to alcohol or drug cues prior to, and during stimulation, enhances clinical outcome for rTMS in alcohol and substance use disorders (Dinur-Klein et al., 2014; Zangen et al., 2021). Overall, this protocol lays the groundwork for a blueprint which can be adapted to rigorously evaluate a broad set of rTMS parameters. Notably, this study evaluated the influence of rTMS on drinking in 66 mice at a relatively low cost and a rapid speed. A clinical trial of the same scale (~65 participants) would require multiple years to acquire funding, collect and interpret data.

While this first-of-its-kind manuscript is a useful blueprint upon which to build future studies, it is notable that the majority of the secondary, experimental results related to changing drinking were not statistically significant. While we detected intriguing trends in changes to drinking behavior, the only drinking result which withstood statistical scrutiny was iTBS increasing alcohol consumption among female mice. There are several reasons we may have observed only modest changes in drinking behaviors within this study. First, we note that the estimated rTMS stimulation strength delivered to the mice (approximately 4 V/m) in this study is lower than in humans (approximately 60–100 V/m) (Padula et al., 2024; McCalley and Hanlon, 2021) and therefore more accurately reflects low-intensity, or sub-threshold stimulation. Further, we note that the electrical field induced by our rTMS coil is relatively broad and may stimulate several regions of the frontal cortex that generate opposing influences on alcohol seeking behaviors (Kravitz et al., 2015). Second, we note that here we delivered 14 sessions of rTMS. While this experimental decision maps closely onto the human rTMS literature for alcohol and substance use disorders (which typically have not yet exceeded 15 rTMS sessions), notable rTMS clinical trials for other psychiatric indications (depression, smoking cessation, anxiety) have observed symptom relief following 20–30 sessions (Zangen et al., 2021; Carmi et al., 2018; George et al., 1995). These factors will be important to address in the future and this work provides a novel set of effect sizes which can be used to power future studies. Future work may consider developing more precise preclinical rTMS coils or evaluating rTMS parameters like the number of sessions.

While the overall influence of rTMS on alcohol consumption within our whole sample was relatively modest, we detected statistically significant effects of sex on change in ethanol consumption following rTMS. First, consistent with the preclinical alcohol literature regarding sex differences and hormonal influences on alcohol drinking in rodents (Priddy et al., 2017; Jury et al., 2017), female mice in our study displayed increased alcohol intake relative to males at baseline, prior to rTMS initiation. Interestingly, we also found that female mice drank significantly more ethanol following iTBS, relative to sham – a finding we did not detect in males. Conceptually, it is possible that delivering iTBS to the rodent fronal cortex increased activity within fronto-striatal, or other reward based cortico-subcortical circuits, which may underlie this increase in alcohol consumption. On the otherhand, cTBS would not necessarily increase this activity given its inhibitory nature and lower impact on electrophysiology (Benali et al., 2011). Future work combining this preclinical rTMS technique with high resolution research tools (e.g. cFOS, optogenetics, DREADDs, etc), may elucidate this relationship further.

In the human literature, sex-differences in clinical and brain-based outcomes following rTMS are of increasing interest, with females broadly experiencing improved clinical outcomes and neural target engagement for a variety of psychiatric disorders (Sackeim et al., 2020; Kedzior et al., 2014; Lisanby et al., 2009; Lefebvre-Demers et al., 2021; Huber et al., 2003) using various rTMS protocols, including iTBS. While the exact mechanisms behind this sex-difference in outcomes remains unclear, potential explanatory variables include differences in skull morphology (which influences distance between TMS coil and cortex, and therefore TMS electrical field strength), differences in grey and white matter integrity, and different densities of sex-hormones like estrogen, progesterone and testosterone (Hanlon and McCalley, 2022). Sex-differences, however, have not yet been rigorously evaluated within rTMS-AUD clinical trials, likely owing to the relatively low census of females within AUD treatment programs and clinical trials (Pinedo et al., 2020; Gunn et al., 2022). Given the finings within this manuscript, and the greater context of emerging sex-differences in the clinical literature, we suggest this preclinical rTMS model will be a value tool to prospectively evaluate sex-diffrences in rTMS-induced changes to alcohol consumption.

A final, substantial advantage in using preclinical models to evaluate rTMS effects on drinking behaviors is the opportunity to examine changes in protein expression which may be related to behavioral change. BDNF expression and genotype has emerged within the literature as a notable biomarker of response to rTMS within clinical populations (Kim et al., 2021). As an experimental innovation, we delivered rTMS to awake, freely behaving mice, thereby circumventing the confounding influence of anesthesia on rTMS-induced change in BDNF expression (Gersner et al., 2011). Here, we observed an interesting finding related to BDNF expression, such that iTBS and cTBS both significantly reduced BDNF expression in mice which consumed only water. Given the general framework that iTBS is ‘excitatory’ or BDNF-promoting, while cTBS is ‘inhibitory’ or BDNF-reducing, this result may be somewhat suprising and begets future investigation. It is possible that delivering rTMS to otherwise healthy, water-drinking mice may illuminate mechanisms behind these non-protocol specific reductions in BDNF expression. There have been few studies specifically investigating the effects of rTMS on healthy mice, however evidence thus far has been mixed. A recent spatial transcriptomics study using a stronger coil but still sub threshold stimulation on 3 month old male mice uncovered the complexities of the brain response to rTMS, such that it is location and and protocol-dependent (Ong and Tang, 2025). Additionally 10 Hz excitatory stimulation in healthy rats with a low-intensity coil increased GABA after 7 days of stimulation but this effect did not persist for 14 days of stimualtion (Seewoo et al., 2019). As our mice received rTMS over the course of 7 days, perhaps timing is a key component in measuring direct downstream effects of rTMS. Therefore, while it is unclear specifically why BDNF decreased following both cTBS and iTBS in our protocol, the complex biological cascades in response to rTMS should be further evaluated in rodent models to better elucidate these components. Further, while no other findings among alcohol drinking mice were statistically significant, it is possible that future work delivering higher doses of rTMS (via increased number of pulses or sessions) may yield greater changes in BDNF expression. On the whole, we highlight that this preclinical model offers the advantage to fill scientitic gaps in our knowledge related to the influence of rTMS on change in gene expression, protein density, and other celluar mechanisms of interest in the context of alcohol consumption.

There are several limitations to the current work which may be expanded upon in future studies. First, the chosen model of alcohol consumption more closely represents human binge drinking rather than AUD or physiological alcohol dependence. Additionally, we did not evaluate temporal dynamics of alcohol consumption or blood-alcohol concentrations following DID, limiting our ability to definitively assess the degree of intoxication or binge-like drinking within each animal. Future work may consider using alcohol vapor chambers or other alternative models of rodent alcohol use which induce physiological alcohol dependence prior to alcohol drinking behavioral assays or rTMS delivery. Another possibility specifically for DID would be 24 hr access to the two-choice bottle for a longer period of days (Giardino et al., 2011), which may better capture changes in drinking preference. Similarly, we evaluated the response of iTBS and cTBS on alcohol consumption only and did not test if this is specific to alcohol or reward-based behavior in general. We aimed to establish an effect based off of our novel protocol and believe that future investigations involving other drugs or sucrose would be valuable.

Another direction that may be addressed in future work is evaluating differences in timing of rTMS. For example, is rTMS most effective in reducing drinking when delivered on the same days as alcohol consumption, during early abstinence, during extended abstinence, or perhaps paired with extinction learning? Future work may consider building upon our model to answer this question, which would likely have direct impact on clinical rTMS delivery for AUD. As noted, due to physical limitations and coil heating, we delivered sub-threshold rTMS intensities. Future research may utilize higher a intensity rodent coil previously shown to induce changes in synaptic densities (Tang et al., 2021) on a similar paradigm to compare differences in biochemical and behavioral effects. Last, we note that our sample size per condition was relatively modest, and the only significant effect of drinking was that of female mice who received iTBS. Given the potential sex differences, future research should include more than n = 6 per subgroup to not only tease apart sex effects but to boost power in the overall study. Given this relatively small sample size, we primarily focus on effect sizes within this manuscript, to provide an informative summary of our work (Sullivan and Feinn, 2012), enhance reproducibility and form a blueprint for future, larger scale studies which may seek to evaluate the influence of rTMS parameters on alcohol consumption.

## Conclusions

To the best of our knowledge, this report represents the first application of rTMS to a preclinical model of alcohol use. This sham-controlled, sex-balanced study observed a significant increase in alcohol consumption among female mice that recived iTBS, relative to sham stimulation. These data warrant future investigation in a well-powered, preclinical analysis designed to assess behavioral change. Overall, we intend for the ‘take-home’ message of this manuscript to be that this model is a feasible, cost-effective, and rapid method to evaluate a variety of factors which may influence rTMS-induced change in alcohol consumption.

## Supplementary Material

Supplementary Materials

Appendix A. Supporting information

Supplementary data associated with this article can be found in the online version at doi:10.1016/j.transm.2025.100098.
