# Supplementary material for: Developing a reverse translational model of low-intensity rTMS in alcohol use disorder: The influence of theta burst stimulation protocols on binge alcohol drinking in mice
Source: Transcranial Magn Stimul. Author manuscript; Available in PMC 2025 Aug 1. (PMC12201979; doi:10.1016/j.transm.2025.100098)
Supplement: Supplementary Materials [file NIHMS2088553-supplement-Supplementary_Materials.docx]

**Developing a reverse translational model of low-intensity rTMS in Alcohol Use Disorder: the influence of theta burst stimulation protocols on binge alcohol drinking in mice**

Akriti Dhungana^1,2^‡, Daniel M. McCalley^1,2^‡, Alesha M. Heath^1^, Eric P. Kraybill^1^, Fatemeh S. Mojabi^1^, Jairelisse Morales Morales^1,2^, Allison R. Morningstar^4^, Allyson K. Davis^1,3^, Claudia B. Padula^1,2^, William J. Giardino^2,4^*, M. Windy McNerney^1,2^*

1. Veterans Affairs Palo Alto Healthcare System, Sierra Pacific Mental Illness Research Education and Clinical Center (MIRECC), Palo Alto, CA
2. Stanford University School of Medicine, Department of Psychiatry and Behavioral Sciences, Stanford, CA
3. Stanford University, Department of Psychology, Stanford, CA
4. Stanford University, Wu Tsai Neurosciences Institute, Stanford CA

**Supplemental Table 1: 2-way ANOVA (sex, treatment) evaluating change in alcohol consumption (A) and preference (B) before and immediately after rTMS.**

**
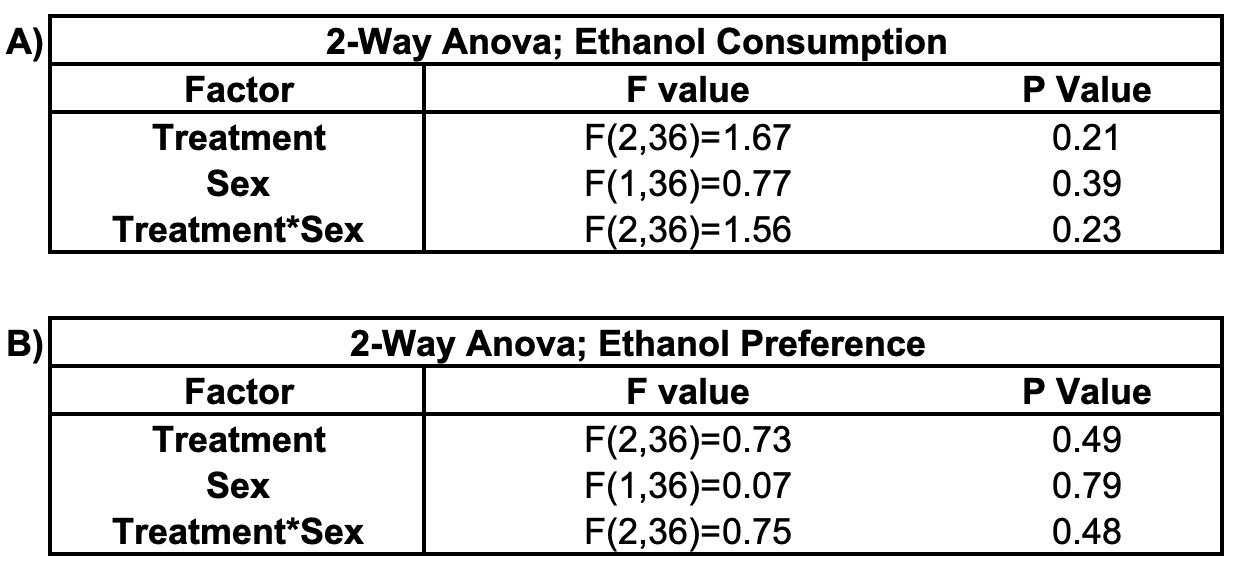
**

**Supplemental Table 1:** A) There were no significant main effects or interactions on change in alcohol consumption. Bruesch-Pagan, p=0.20; Leven’s test, p=0.38. B) There were no significant main effects or interactions on change in alcohol preference. Bruesch-Pagan, p=0.48; Leven’s test, p=0.24.**Supplemental Table 2: 3-way repeated measures ANOVA (time, sex, treatment) evaluating change in alcohol consumption (A) and preference (B) at 3 time points: 2 days pre-rTMS, 2-days immediately post-rTMS, and days 3 and 4 post-rTMS.**

**
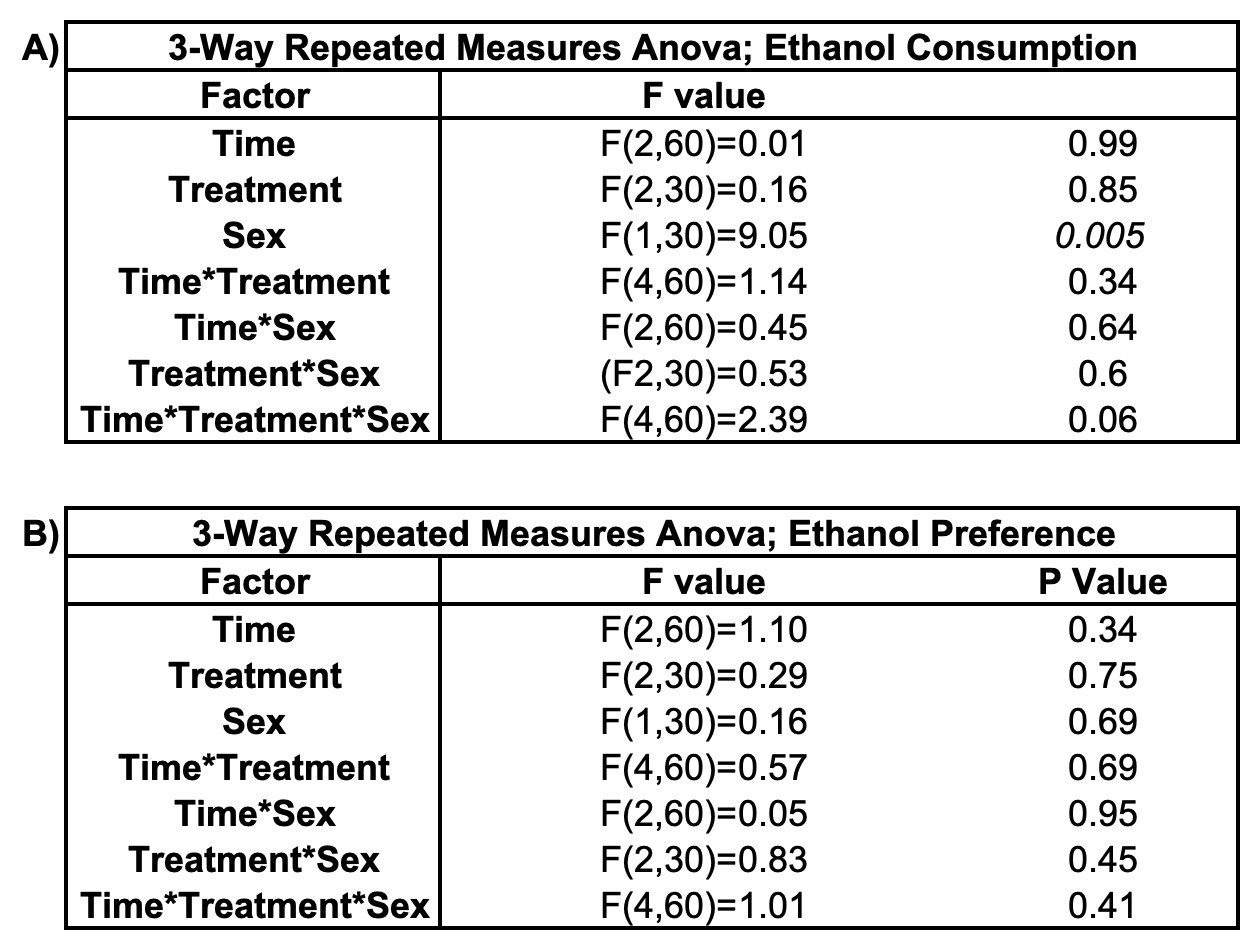
**

**Supplemental Table 2:** A) There was a main effect of sex (p=0.005) and a trend-level time*treatment*sex interaction (p=0.06) on alcohol consumption within the experiment. Levene’s test, p=0.11; Mauchly’s test for sphericity, p=0.34. B) There were no significant main effects of interactions on alcohol preference within the experiment. Levene’s test, p=0.58; Mauchly’s test for sphericity, p=0.73.

**Supplemental Figure 1: Baseline Sex-difference in EtOH and water consumption**

**
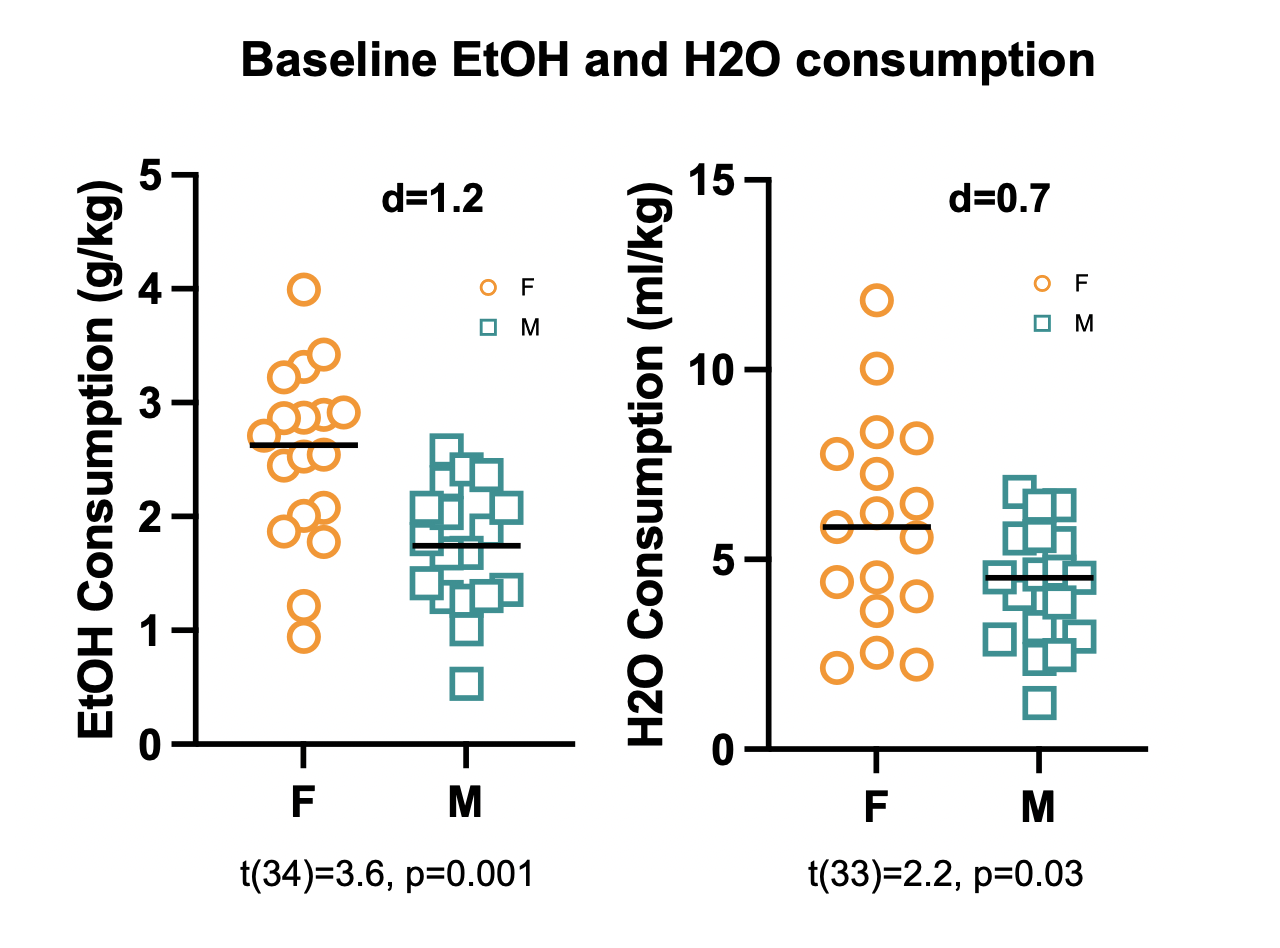
**

**Supplemental Fig. 1:** Females (orange circles) drank more ethanol (left) and water (right), relative to males (teal squares) during the baseline DID assessments. There was no overall difference in preference for alcohol between sexes (Female: 72.75 ± 13.4%; Male: 72.12 ± 11.2%). Black lines represent sample median.

**Supplemental Fig 2: Average ethanol intake and preferences pre and post rTMS**

**B)**

**A)**

**Supplemental Fig. 2:** A) Ethanol intake B) and Preferences averaged for the two days prior to rTMS (Pre 6-7), the first two days following rTMS (Post 1-2), and then the average of days 3-4 Post rTMS (Post3-4) for females (F, light circles) and males (M, dark circles) by treatment condition. The average was taken for these days to account for any left vs right side preferences for ethanol bottle placement in the cage.

**Supplemental Fig. 3: Change in water consumption among animals only exposed to water.**

**
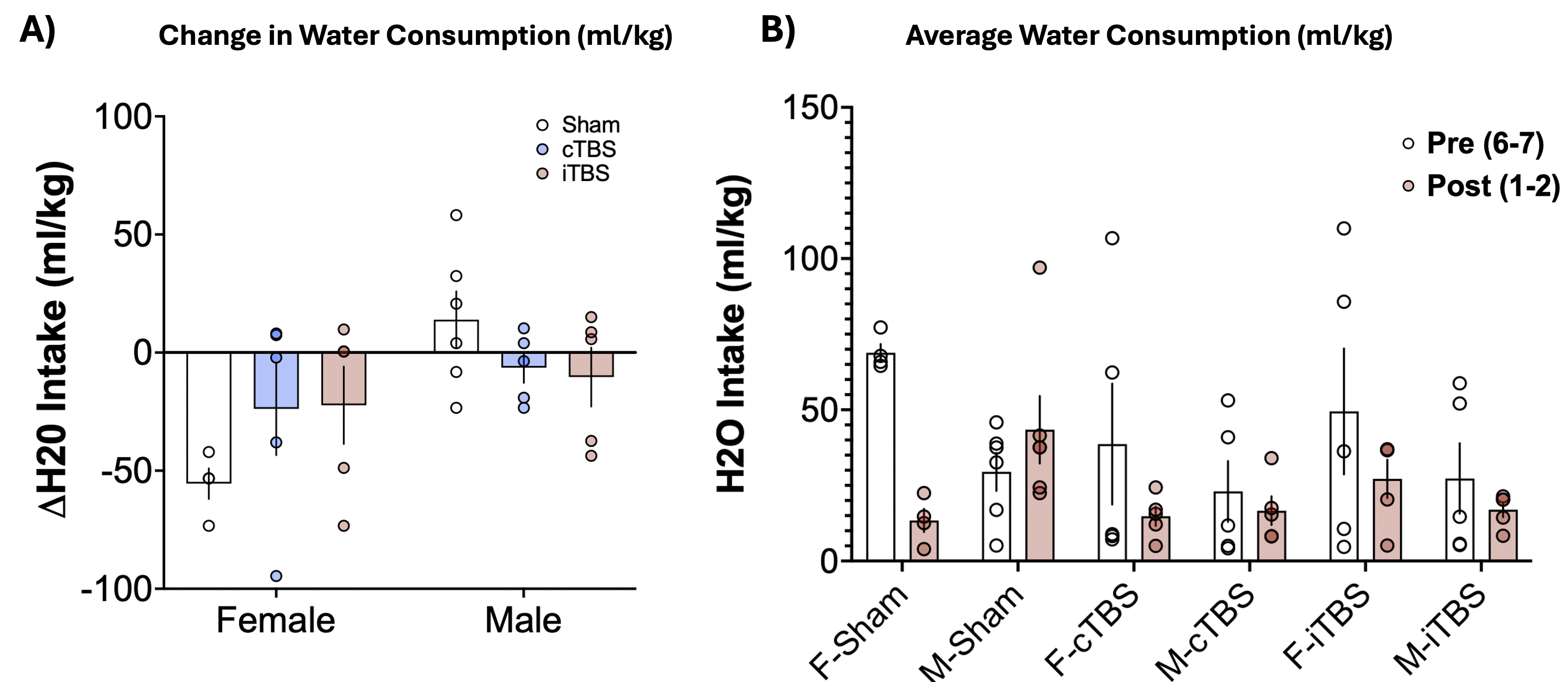
**

**Supplemental Fig. 3:** A) Female animals which were only given water during DID drank less water following rTMS and sham stimulation. Male animals maintained consistent water drinking pre and post rTMS. B) Average water consumption before (white bars) and after (red bars) rTMS. Error bars reflect standard error of the mean. Individual data points are plotted with each circle.

**
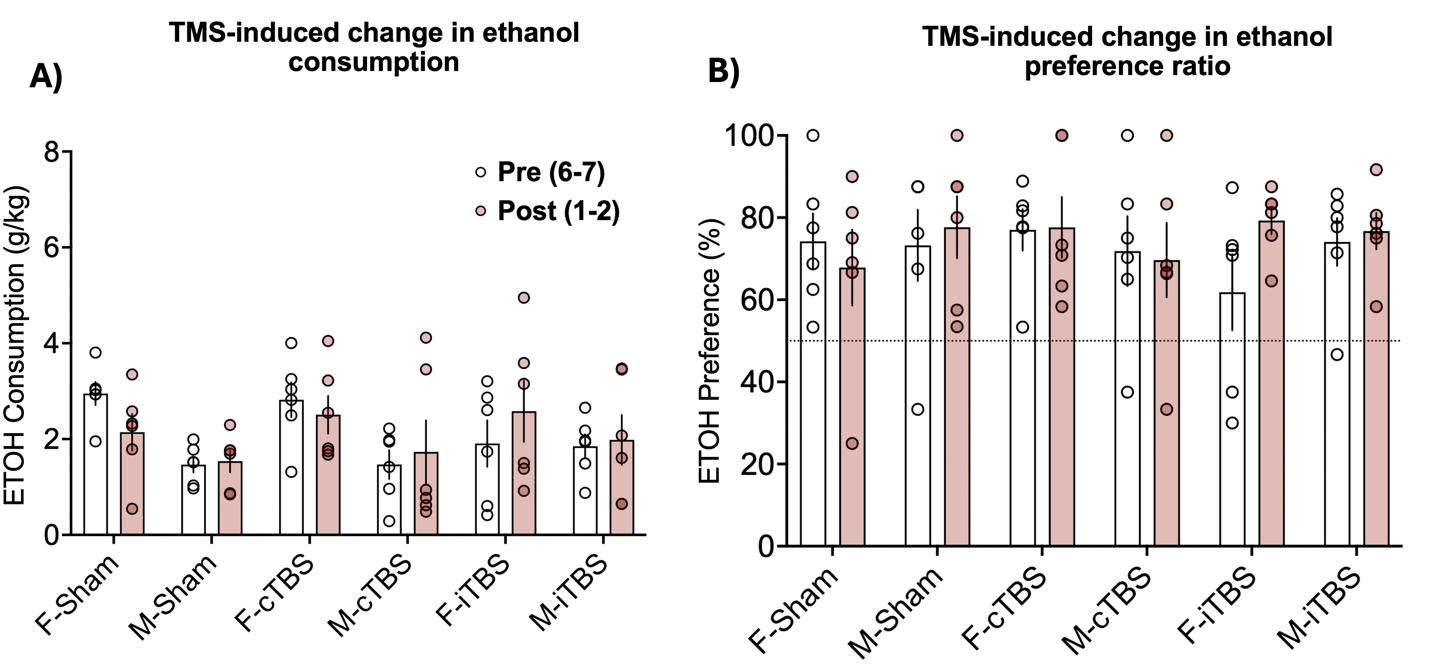
Supplemental Fig 4: Average ethanol consumption and preference before and after rTMS**

**Supplemental Fig. 4** A) Average ethanol consumption (g/kg) before (white bars) and after (red bars) rTMS. B) Average ethanol preference before and after rTMS. Dotted line represents 50%, or equal preference for ethanol and water. Error bars reflect standard error of the mean. Individual data points are plotted with each circle.

**Supplemental Fig. 5: Sex differences in BDNF expression (normalized to same-sex H20-sham animals)**


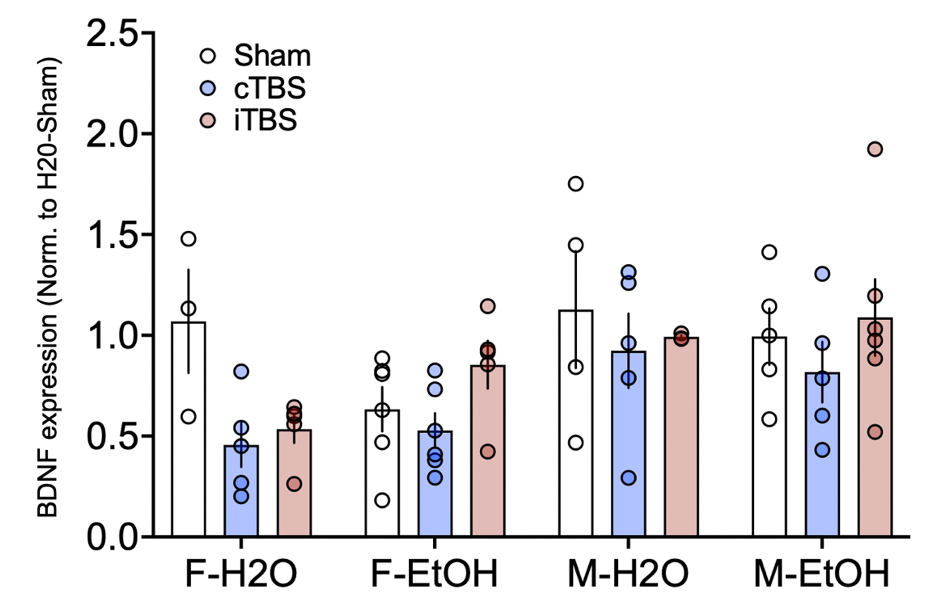


**Supplemental Fig. 5:** BDNF expression levels following rTMS were consistent with Figure 5 when normalized to same-sex, water drinking only, sham animals. Error bars reflect standard error of the mean. Individual data points are plotted with each circle.
